# Supplementary material for: Dynamic Prediction of Mechanical Thrombectomy Outcome for Acute Ischemic Stroke Patients Using Machine Learning
Source: Brain Sci. 2022 Jul 18;12(7):938. doi: 10.3390/brainsci12070938 (PMC9313360; doi:10.3390/brainsci12070938)
Supplement: Supplementary file 1 [file brainsci-12-00938-s001.zip › brainsci-1796903-supplementary.pdf]

Supplementary Table S1. Hyper-parameters of each XGBoost model.

Model	Hyper-parameters	parameter values
“Admission” model	n_estimators	130
	max_depth	5
	min_child_weight	6
	Seed	2
	gamma	0.1
	colsample_bytree	0.4
	subsample	0.4
	reg_alpha	0
	reg_lambda	1
	learning_rate	0.06
“24-Hour” model	n_estimators	100
	max_depth	8
	min_child_weight	5
	Seed	0
	gamma	0.1
	colsample_bytree	0.6
	subsample	0.6
	reg_alpha	0
	reg_lambda	1
	learning_rate	0.05
“3-Day” Model	n_estimators	100
	max_depth	8
	min_child_weight	5
	Seed	0
	gamma	0.1
	colsample_bytree	0.6
	subsample	0.6
	reg_alpha	0
	reg_lambda	1
	learning_rate	0.05
“Discharge” model	n_estimators	100
	max_depth	8
	min_child_weight	3
	Seed	1
	gamma	0.1
	colsample_bytree	0.55
	subsample	0.55
	reg_alpha	0
	reg_lambda	1
	learning_rate	0.06

Abbreviations: XGBoost, eXtreme gradient boosting.

Supplementary Table S2. The p-values of pairwise comparisons of AUCs on the testing set for different models with the Delong test.

Model	"24H" model	"3Days" model	"Discharge" model	THRIVE	HIAT
"Admission" model	0.011	0.081	0.015	0.003	0.012
"24H" model		0.235	0.021	0.001	0.008
"3Days" model			0.139	0.002	0.003
"Discharge" model				0.001	0.002
THRIVE					0.950

The significant difference between AUCs is defined as *p*-value < 0.05.

Abbreviations: AUC, the area under receiver operating characteristic curve; THRIVE, Totaled Health Risks in Vascular Events; HIAT, Houston Intra-arterial Recanalization Therapy.

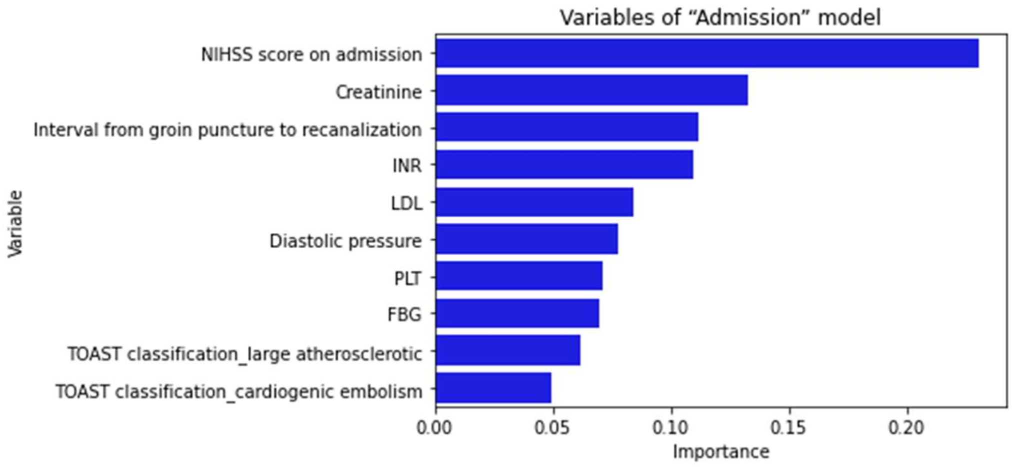

(a)

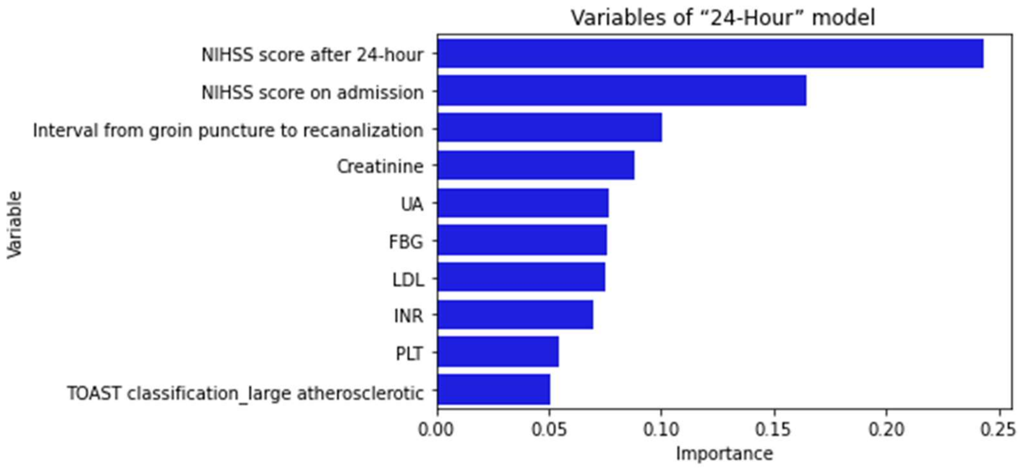

(b)

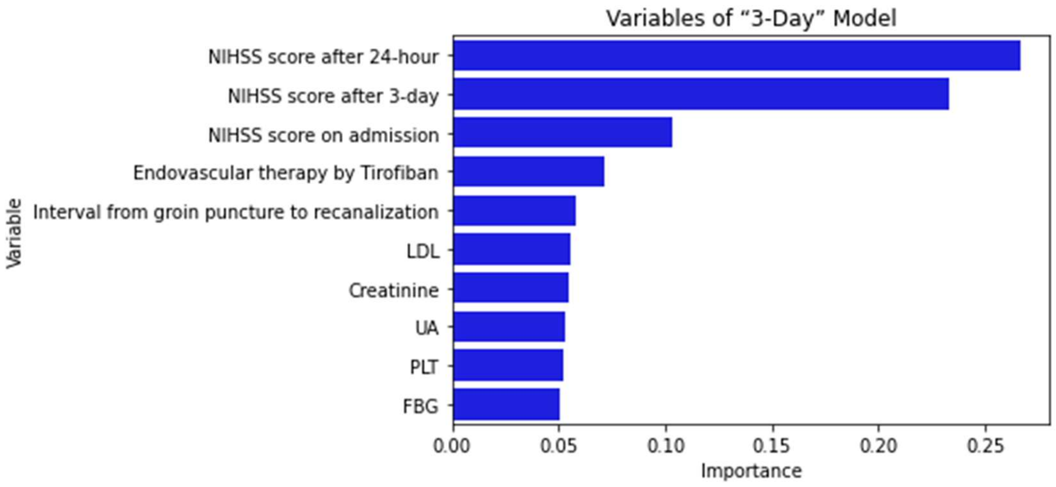

(c)

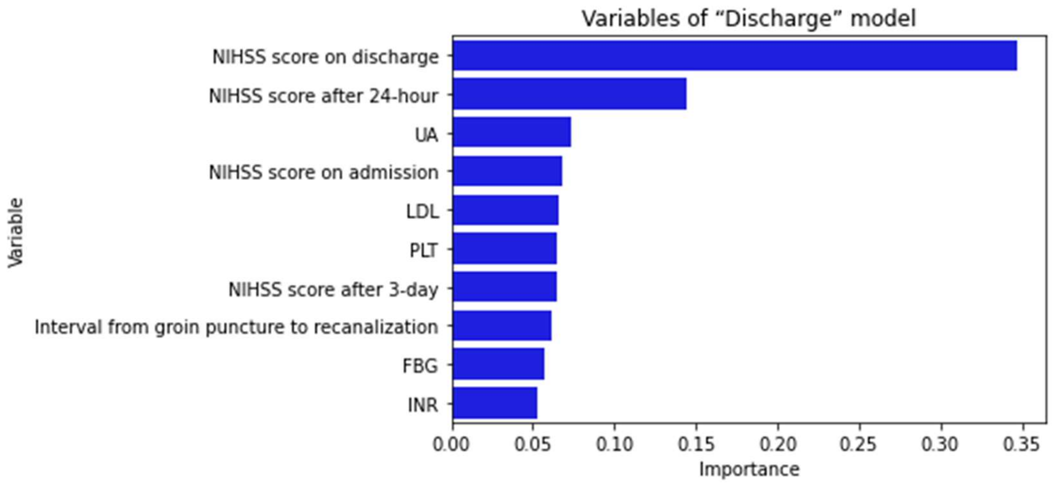

(d)

Supplementary Figure S1. (a) Top 10 features selected using eXtreme gradient boosting (XGBoost) and the corresponding variable importance score in “Admission” model; (b) Top 10 features selected using XGBoost and the corresponding variable importance score in “24-Hour” model; (c) Top 10 features selected using XGBoost and the corresponding variable importance score

in “3-Day” model; (d) Top 10 features selected using XGBoost and the corresponding variable importance score in “Discharge” model. X-axis indicates the importance score which is the relative number of a variable that is used to distribute the data, Y-axis indicates the top 10 weighted variables. NIHSS, National Institute of Health stroke scale; INR, International normalized ratios; LDL, Low density lipoprotein; PLT, Platelet; FBG, Fasting blood glucose; TOAST, Trial of ORG 10172 in Acute Stroke Treatment; UA, Uric Acid
